# Control of internal validity threats in a modified adaptive platform design using Halili Physical Therapy Statistical Analysis Tool (HPTSAT)

**DOI:** 10.1016/j.mex.2021.101232

**Published:** 2021-01-15

**Authors:** Adi Halili

**Affiliations:** Halili Physical Therapy, 268 E River Rd. #130, Tucson, AZ 85704, United States

**Keywords:** Systemic manual therapy, Internal validity, HPTSAT, Adaptive platform model, PREVAIL II, Physical therapy, Statistical analysis

## Abstract

Some of the challenges in evaluating the effectiveness of physical therapy practice stem from the common lack of interventional standardization, as well as problems with both the availability and routine use of specific, valid outcome tools. But even if these issues are dealt with, there are still significant validity threats when trying to understand the effectiveness of physical therapy interventions.

Among the most critical internal validity threats: repeated testing effect, study sample uniformity, and increase in type I or type II error.

The purpose of this analysis is to illustrate how these internal validity threats were controlled using the Halili Physical Therapy Statistical Analysis Tool (HPTSAT).

The original concept behind the HPTSAT tool was the adaptive platform design used in the PREVAIL I and II protocols(1,2,3). However, this concept has been significantly expanded upon in the HPTSAT design to allow for the simultaneous analysis of hundreds of treatment combinations while controlling for the above-mentioned critical internal validity threats.

• HPTSAT allows for concurrent computerized analysis modeled and improved upon the adaptive platform design used in the PREVAIL I and II protocols.
• This analysis is possible because the tool isolates the average rate of change (ARC5) instead of average change after treatment.
• This paper provides the mathematical basis for the algorithm used in the tool to control for several internal validity threats including repeated testing effect, study sample uniformity, and increase in type I and II error.

## Graphic abstract

**Figure:**
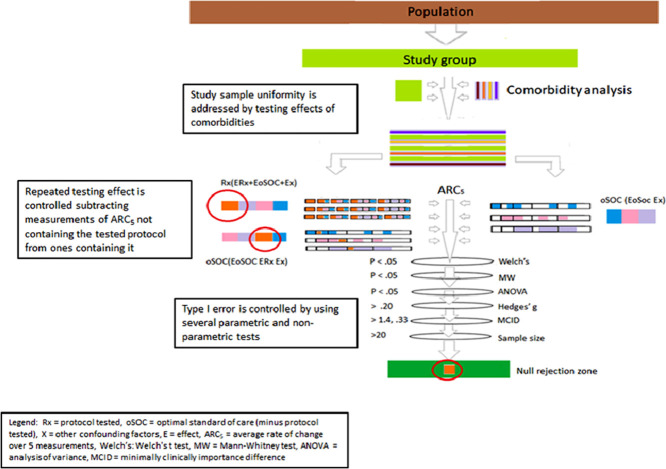

Specifications table

**Table:** 

Subject Area:	Medicine and Dentistry
More specific subject area:	Physical Therapy, Systemic Manual Therapy
Method name:	Halili Physical Therapy Statistical Analysis Tool (HPTSAT)
Name and reference of original method:	NA
Resource availability:	Email requests to halilipt@msn.com

## Methodology

This study used the HPTSAT to import and blind treatment records from the Halili PT 2015 database (HPT2015) [6]. In the HPT2015, all physical therapy treatment visits were recorded, and outcome tools were used each visit.

The challenges posed by interventional standardization and the use of outcome tools were addressed in the HPT2015 database before importation to the HPTSAT and are not technically part of this analysis. However, it is important to briefly discuss how these two issues were handled.

Interventional standardization occurred since most treatment sessions were done using defined standardized treatment protocols [4]. Notation of each protocol was done using a 2 to 5 letter code to allow for the subsequent computerized analysis.

The standard application of treatment was possible because of several unique attributes of the practice setting. First, most interventions were provided using methods such as Barral [10], strain counterstrain [7] or fascial counterstrain [8], integrative manual therapy [9], and muscle energy techniques [11]. The common thread behind all these methods is that the techniques are done in the same manner in each application. Moreover, specific to the practice setting investigated, individual techniques were grouped into distinct protocols which were also performed in the same manner each time [4].

The primary outcome tool used each visit was the Patient Identified Problem scale (PIP) [5]. The PIP scale is a 1 to 10 (half point permitted) scale. The patient can score between 1 (which denotes that the problem is not currently active) to 10 (indicating maximal intensity). The problems are looked at both individually and as a cumulative score.

The PIP scale has specificity and sensitivity of 91.46% and 64.45%, respectively, and an ICC score of 0.96. MCID for change observed in the whole scale is 3.8 (95% CI 1.4 to 8.2), and for a specific problem, the score change is 0.89 (95% CI 0.33 to 1.5) [5].

The initial concept behind the analysis method used in this study was the adaptive platform design. This research model came into light with the PREVAIL I and II protocols for the Ebola virus (EBV) outbreak in 2014 [1], [2], [3]. Under this design, a concurrent evaluation of several different investigational agents was allowed. This strategy's concept was that several agents were tested simultaneously against a control group that received the otherwise optimal standard of care (oSOC). Once an agent was deemed effective, it would become part of the new oSOC to be tested against other new agents.

When the term “investigational agents” is substituted for “treatment protocols” used in the practice, we could see a similar endpoint where multiple interventions comprise a new oSOC.

The critical difference between the PREVAIL protocols and HPTSAT is that while in the PREVAIL model, the adaptation of oSOC and addition of new agents were made incrementally, in the HPTSAT, the observations are done retrospectively and in a simultaneous manner.

The HPTSAT performed an automated analysis comparing each combination of 1,2,3,4 and 5 protocols (frequency >5) to all the remaining protocol combinations. The tested protocol or protocol combination is referred to in this discussion as the Rx, and the remaining combination group is the oSOC.

While there are inherent advantages to using an oSOC that includes all other treatment protocols and not just the ones that were available at the time a particular agent was tested, there are several internal validity threats we need to control for:

• Repeated testing effect (the effects of treatments are blended because treatments are provided in close temporal proximity).
• Study sample uniformity (patients respond differently to intervention because of their comorbidities).
• Increase in type I error (measuring the difference between groups when there is none)
• Increase in type II error (not measuring difference when one exists).

*Repeated testing effect threat* occurs when different treatments are provided consecutively. The challenge measuring the effects of treatment is that the effects of one treatment bleed into the next measurement point, and what is measured is a combination of effects from several treatments and other confounding factors.

To be able to demonstrate the difference between the treatment effect (E_Rx_) from other treatments (E_oSOC_) and other confounding factors (E_X_), we needed to develop a strategy that would yield a test statistic describing that difference.

**Fig. 1:**
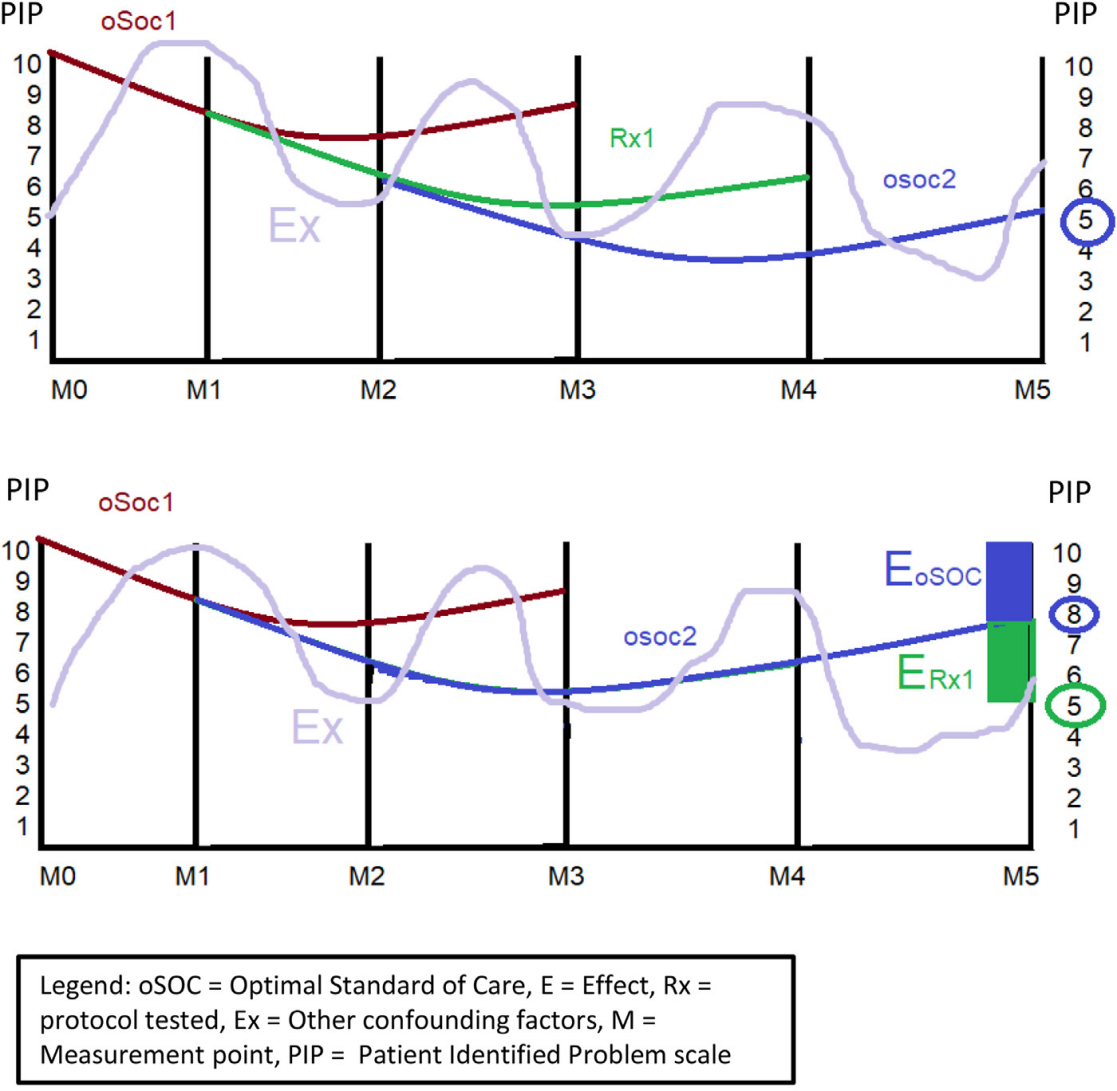
Illustrates this process.

The challenge in coming up with this number is that the E_Rx_ persists over several measurement points (M). Therefore, the analysis needed to include several measurements points to capture the full E_Rx_ over time. Also, a similar number of measurement points were needed to capture the full effects of E_oSOC_ and E_x_. It is the difference between these two observations that allows us to isolate the E_Rx_ (Fig. 1) illustrates this process.

The strategy to separate the effect of the protocol evaluated (E_Rx_) from the effects of other treatments (E_oSOC_) and other confounding factors (E_X_) was to group several measurements (taken before each consecutive visit after a protocol was performed) and compare each to the pre-treatment score. To isolate the E_Rx_ component, this group of measurements was to be compared to a similar group that did not contain the tested protocol, hence containing only E_oSOC_ and E_X_.

Because what we were testing was not the difference between two points, but rather the change over several measurements’ points, the test statistic we identified was the average rate of change (ARC).

However, before this type of analysis could be done for pragmatic design reasons, we needed an approximation of the range of time it takes for a treatment effect (E) to fully occur.

To do so, using the HPTSAT, we sampled average treatment changes measured at an interval between 2 and 40 days after treatment was provided. We did so during several test analyses for various problems. The results allow us to compute a Mean and 95% CI of the top 25% of the scores, providing a peak effect for most tests somewhere between 10 and 18 days with 95% CI ranging ± 5–7 days.

Once we established this rough estimate, we could reasonably determine that if we program the HPTSAT algorithm to take up to 5 measurements after a treatment protocol is provided, we will capture all or most of the effect E_Rx_. Because of this last observation, when reporting the test statistic, HPTSAT was programmed to use the term ARC_5_ to denote the number of automated measurements per protocol.

Naturally, there are no guarantees that all future peak effects would be captured in the five measurement points. To address that, HPTSAT would run the peak effect test before each automated analysis. If the 95% CI of that effect goes beyond the five measurement points, there is a possibility of a Type II measurement error (not measuring an effect when one exists). The handling of this error is discussed later in this paper.

Next, HPTSAT isolated the average rate of change (ARC_5_), measuring change rates for individual problem and overall PIP scale change rate for each protocol or protocol combination performed in a frequency of 5 or more on the patients in the study group.

**Fig. 2:**
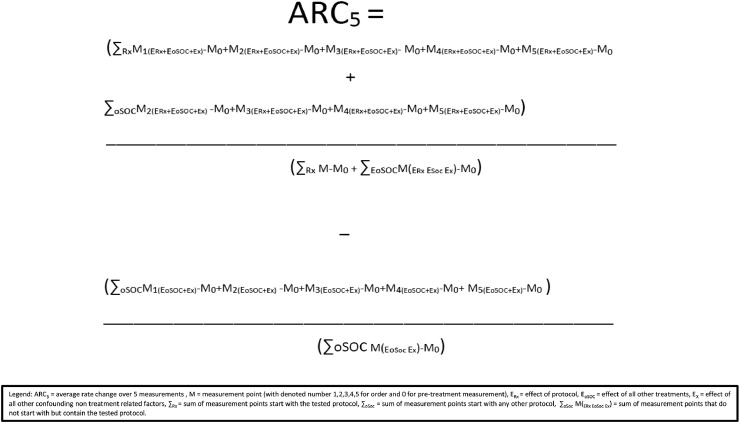
Average rate of change computational formula.

Fig. 2 include the formula illustrating the comutation for each protocol or protocol combinations.

Where: ARC_5_ = Average rate change over five measurements, M = measurement point (with denoted number 1,2,3,4,5 for order and 0 for pre-treatment measurement), E_Rx_ = effect of protocol, E_oSOC_ = effect of all other treatments, E_X_ = effect of all other confounding non-treatment related factors, ∑_Rx_ = sum of measurement points start with the tested protocol, ∑_oSoc_ = sum of measurement points start with any other protocol, ∑_oSoc_ M(_ERx EoSoc Ex_) = sum of *measurement* points that do not start with but contain the tested protocol.

Because five measurements are taken, and we previously estimated that most treatment E_Rx_ rate of change would zero out after three measurements (based on the peak effect test), the E_Rx_ can be fully captured even when measuring the effects of a group of up to 3 protocols together. When measuring the ARC_5_ of combinations containing 4 or 5 protocols, there is a small chance for a type II measurement error because of the possibility that the E_Rx_ of the 4th or 5th protocol in the sequence would not be fully captured in the M_5_ measurement. The handling of this issue is also discussed later in this text.

Table 1 illustrates how HPTSAT displays the results of this analysis (the number of samples shown represents only a small fraction of the actual analysis and is presented for illustration purposes only). The dark green shading indicates that the sample passed all pre-specified null rejection criteria (sample size larger than 20, ARC_5_ larger than MCID low value of 95% CI, Welch's, Mann-Whitney (MW), ANOVA *p* < .05, and Hodges’ *g* > 0.2). The pink shading indicates items that failed the criteria).

**Table 1:** Results by overall PIP scale.

*Study sample uniformity*: The second internal validity threat to be handled by HPTSAT is the study sample's undetermined uniformity. This threat was handled in the following manner: During the automated portion of the analysis, HPTSAT evaluated the difference across an episode of care (the difference between PIP or individual score before the first treatment compared to the score recorded on after the last treatment). Then that analysis was repeated in the presence and absence of 12 factors: gender female, gender male, history of abdominal surgeries, bowel/bladder/reproductive problems, cancer, cardio/respiratory issues, insomnia, metabolic disease, neurological surgeries, neurological conditions, orthopedic surgeries, and history of smoking and/or alcohol use. This analysis was done when a sample size larger than ten was available for comparison in the presence and absence groups (ensuring that the subsample is ≥20). The significance of the difference was measured using Welch's *t*-test, Mann–Whitney test, Hodges’ *g*, and ANOVA for effect size.

Post-hoc testing also allowed for evaluation of the above comorbidities across any protocol sequence (maximum of 5) or individual protocols.

The post-hoc testing cache also allows for testing for the effect of additional comorbidities across an episode of care, including other patient-identified problems (for example, if the patient-tested complaint was lower back pain, the test can assess the effect of also having leg pain). Other capabilities include the ability to test the effect of medication or medication combinations (up to 3) and the effect of a selected age range (for example, patients between the ages of 70 and 90 would fare differently from the rest of the study sample). Beyond the obvious clinical interest in understanding the effects of these comorbidities, this information allows development of a clinical prediction rule as to which patient profile would fare best with a given treatment, hence reducing the dependance on sample homogeneity for data interpretation.

Table 2 is an example of how these results are displayed in HPTSAT (The number of samples shown represents only a small fraction of the actual analysis and are presented for illustration purposes only). The fields shaded green indicated that the difference between the groups had a *p* < .05 for Welch, MW, and the ANOVA tests. The red shading indicates a statistically significant detrimental effect on the outcome, and light blue shading indicates a statistically significant beneficial effect on the outcome. The pink shading indicates a failure to pass the preset null rejection criteria for that specific test or benchmark.

**Table 2:** Effects of comorbidities, other pip complaints or medications on PIP scale.

*Type I error:* The third internal validity threat that needs to be addressed by HPTSAT is the risk of an increase in type I error due to irregular sub-sample characteristics. This concern arises from the fact that during the automated and post-hoc tests, there are hundreds of samples being evaluated, and without further analysis, outliers and distribution characteristics of these subsamples are unknown.

While it is impossible to eliminate this threat by prospective design measures since this evaluation is done retrospectively, we did take several measures to identify when this error is likely to occur and what outcomes we need to interpret with caution. These measures included establishing null rejection criteria (asserting a difference between the two samples). These criteria included: sample size above 20, ARC_5_ score above the lower score of the PIP scale MCID 95% CI, Welch's *t p* < .05, MW *p* < .05, ANOVA *p* < .05 and Hedges’ *g* >0.2.

Choosing a sample size above 20 increased the chances that this sample would be normally distributed. Choosing Welch's test over the Student *t*-test allows handling of a sample pair with unequal variances. The addition of ANOVA and Hedges’ *g* allows for evaluating other parametric factors such as variances and standard deviation, respectively. The use of the MW test allows for the evaluation of non-parametric factors such as ranked medians. Also, the decision to use Hedges' *g* over Cohen *d* was made due to the former's better handling of unequal sample size.

See Table 1 for an example of how these tests are displayed in the results.

*Type II error:* is probably the least clinically important among the threats discussed so far. The reason is that in practice, the existence of this error is only meaningful for a small number of samples. Samples that passed the null rejection criteria would still pass this criterion even when the error is not corrected. Samples that were rejected by a sizable number are not likely to pass after correction because the correction is likely to be exceedingly small. To understand the reason for this, we need to consider the two scenarios where this type of error is likely to be seen. The first is when we test a protocol combination of 4 or 5 protocols, and the second is where the peak effect and distribution test indicate that it is likely to take place outside of the M5 measurement. Under both scenarios, most of the treatment effect is still likely to fall within the 5-measurement range. Therefore, the only scenario where it is clinically useful to use the adjustment formula below is when the sample tested is close enough to pass the rejection criteria for the adjustment to make a difference.

To adjust five protocol combinations, HPTSAT allows us to do the following:

Where: E_(Rx12345-1234)_ = ∑E_Rx(12345)_ - ∑E_Rx(1234)_

If √(E_Rx(12345-1234)_)^2^ > √(E_Rx5_)^2^ no adjustment is possible*

If √(E_Rx(12345-1234)_)^2^ < √(E_Rx5_)^2^ the following adjustment should provide a reasonable approximation of the true effect: ARC_5_ = ∑E_Rx(12345)_ – (∑E_Rx(12345)_ - ∑E_Rx(1234)_) + E_Rx5_ + 0.33(E_Rx4_)

To adjust four protocol combinations, we do the following:

Where: E(_Rx1234-123)_ = ∑E_Rx(1234)_ - ∑E_Rx(123)_

If √(E_Rx(1234-123)_)^2^ > √(E_Rx4_)^2^ then no adjustment is possible*

If √(E_Rx(1234-123)_)^2^ < √(E_Rx4_)^2^ then the following adjustment should provide a reasonable approximation of the actual effect:

ARC_5_ = ∑E_Rx(1234)_ – (∑E_Rx(1234)_ - ∑E_Rx(123)_) + E_Rx4_

(* The reason no adjustment is possible is due to a synergy effect that could occur when performing the protocol sequence. This effect makes the overall ARC_5_ larger when done in a particular sequence than the sum of the individual ARC_5_ of the individual protocols done in isolation.)

HPTSAT automatically computes and insert the correction for all 4 and 5 combinations that do not have a synergistic effect. All the corrected combinations are also displayed with the degree of correction in a separate report.

**Fig. 3:**
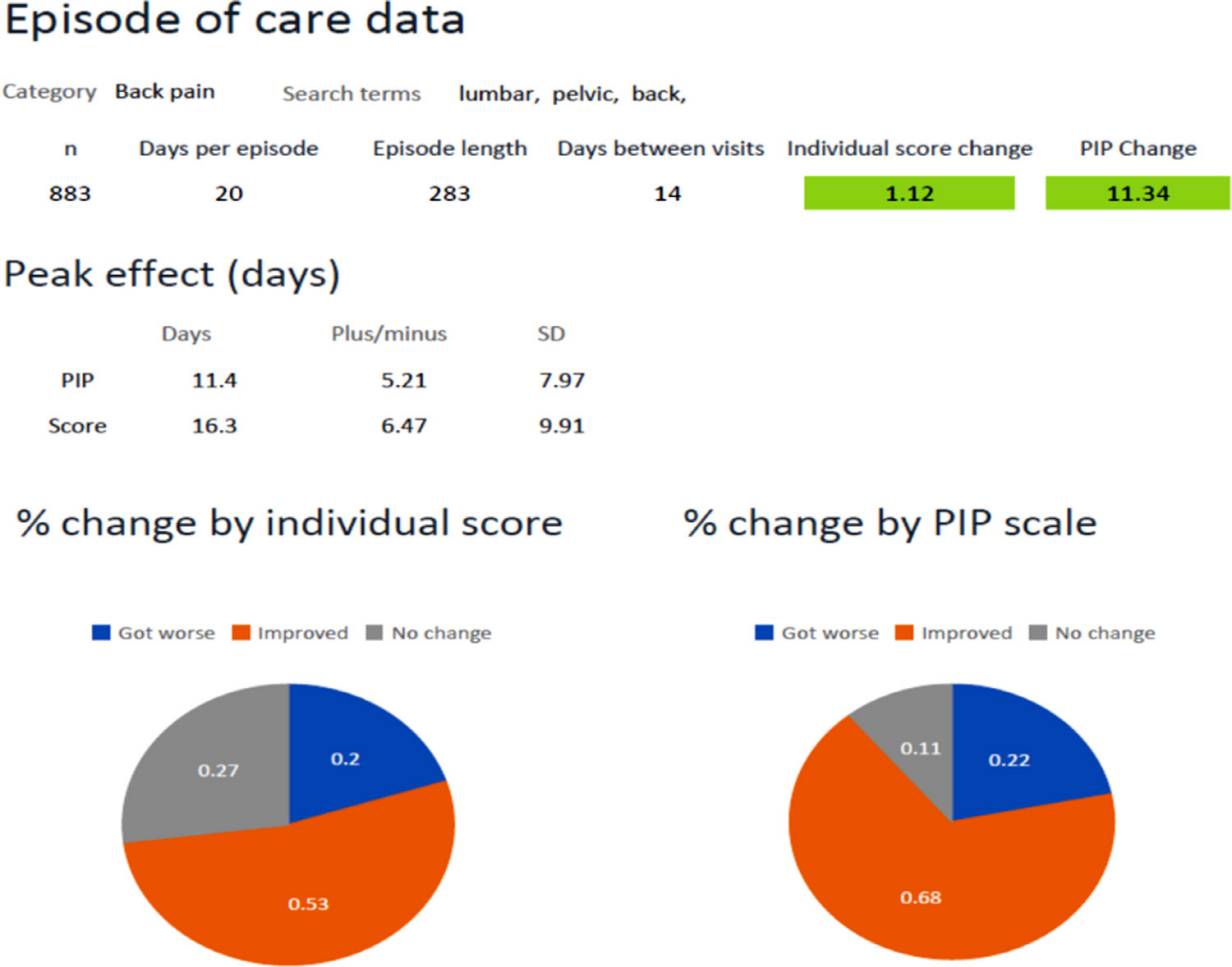
Illustrates how that part of the analysis data is displayed.

In addition to the tests described above, HPTSAT also allows for a more general analysis of changes across an episode of care, rates of improvements, and other comparisons between the study sample and population characteristics. (Fig. 3) illustrate how that part of the analysis is displayed.

## Summary

The HPTSAT offers the researcher the ability to analyze hundreds of treatment combinations comprised of thousands of individual techniques. It allows for general understanding as to how well a specific clinical problem is addressed across an episode of care; it also gives the researcher the ability to extensively compare a large number of interventional strategies. Furthermore, as discussed in this analysis, it can do so while controlling or identifying the major internal validity threats.

## Limitations

This tool can only be used when treatment is provided in a standardized manner, and when a valid and specific outcome tool such as the PIP scale is used.

## Declaration of Competing Interest

The authors of this study have no financial interest or conflict to declare.
